# Outcomes of possible and probable rheumatic fever: A cohort study using northern Australian register data, 2013–2019

**DOI:** 10.1371/journal.pgph.0002064

**Published:** 2024-01-03

**Authors:** Laura Goddard, Mirjam Kaestli, Enes Makalic, Anna P. Ralph

**Affiliations:** 1 Global and Tropical Health Division, Menzies School of Health Research, Charles Darwin University, Darwin, Northern Territory, Australia; 2 School of Global and Population Health, University of Melbourne, Melbourne, Victoria, Australia; 3 Research Institute for the Environment and Livelihoods, Charles Darwin University, Darwin, Northern Territory, Australia; University of Melbourne, AUSTRALIA

## Abstract

In Australia, there is a high burden of acute rheumatic fever (ARF) among Aboriginal and Torres Strait Islander peoples. Clinical diagnostic criteria can result in a diagnosis of ‘definite’, ‘probable’ or ‘possible’ ARF and outcomes range from recovery to severe rheumatic heart disease (RHD). We compared outcomes by ARF diagnosis, where the main outcome was defined as disease progression from: possible to probable ARF, definite ARF or RHD; probable to definite ARF or RHD; or definite ARF to definite ARF recurrence or RHD. Data were extracted from the Northern Territory RHD register for Indigenous Australians with an initial diagnosis of ARF during the 5.5-year study period (01/01/2013–30/06/2019). Descriptive statistics were used to describe cohort characteristics, probability of survival, and cumulative incidence risk of disease progression. Cox proportional hazards regression was used to determine whether time to disease progression differed according to ARF diagnosis. Sub-analyses on RHD outcome, clinical manifestations, and antibiotic adherence were also performed. In total there were 913 cases with an initial ARF diagnosis. Of these, 92 (13%) experienced disease progression. The probability of disease progression significantly differed between ARF diagnoses (p = 0.0043; log rank test). Cumulative incidence risk of disease progression at 5.5 years was 33.6% (95% CI 23.6–46.2) for definite, 13.5% (95% CI 8.8–20.6) for probable and 11.4% (95% CI 6.0–21.3) for possible ARF. Disease progression was 2.19 times more likely in those with definite ARF than those with possible ARF (p = 0.026). Progression to RHD was reported in 52/732 (7%) of ARF cases with normal baseline echocardiography. There was a significantly higher risk of progression from no RHD to RHD if the initial diagnosis was definite compared to possible ARF (p<0.001). These data provide a useful way to stratify risk and guide prognosis for people diagnosed with ARF and can help inform practice.

## Introduction

In Australia, high burdens of acute rheumatic fever (ARF) and rheumatic heart disease (RHD) occur among Aboriginal and Torres Strait Islander peoples, particularly across northern and central Australia [1]. There is no diagnostic test for ARF. This impairs case detection and opportunities for early intervention with antibiotic secondary prophylaxis [2]. The Jones Criteria are syndromic criteria to diagnose ARF, dividing the clinical features into major and minor manifestations based on their prevalence and specificity [3]. The Jones Criteria have been regularly revised in response to evolving clinical knowledge and epidemiology, to reduce overdiagnosis in high-resource settings where ARF has become uncommon [4–6]. As a result, they became less sensitive for detection of cases in high-incidence populations. Different considerations were therefore added for high-risk groups [3,5,6], but to further avoid missed diagnoses in high-prevalence Aboriginal communities in Australia, additional diagnostic categories of ‘possible’ and ‘probable’ ARF were proposed [7]. These categories were further clarified in the revised 2020 Australian Guidelines (Box 1) [3]. This is in contrast to the Revised Jones Criteria, that only articulate two categories (definite and probable ARF), but consistent with other countries requiring highly sensitive criteria in order not to miss cases in populations where there is a high pre-test probability, such as New Zealand [6,8].

**Box 1. Acute rheumatic fever diagnostic categories, 2020 Australian Guidelines [6]**

**Definite ARF**: acute presentation which fulfils the 2015 Revised Jones diagnostic criteria for ARF.

**Probable ARF**: acute presentation which does not fulfil Jones diagnostic criteria for ARF, missing one major or one minor criterion or lacking evidence of preceding streptococcal infection, but ARF is still considered the most likely diagnosis.

**Possible ARF**: acute presentation which does not fulfil Jones diagnostic criteria for ARF, missing one major or one minor criterion or lacking evidence of preceding streptococcal infection, and ARF is considered uncertain but cannot be ruled out.

In Australia’s Northern Territory (NT), definite ARF is a notifiable disease. Since 1997, all notified cases of ARF (including recurrent episodes) and RHD among people living in the NT have been recorded in the NT RHD Register (“the Register”), which was designed to facilitate long term care coordination by primary health providers [9]. Previous research using data from the Register has described disease progression from definite ARF to RHD and other outcomes such as morbidity and mortality [10,11]. In the NT, the risk of disease progression from definite ARF to RHD is high, with a cumulative incidence of progression of 27.1% at 1 year after diagnosis, 44.0% at 5 years and 51.9% at 10 years [11]. Adherence to secondary penicillin prophylaxis (regular injections of benzathine benzylpenicillin) to prevent ARF recurrences reduces risk, with a dose-dependent effect [12]. Possible (and sometimes, probable) ARF has been excluded from previous studies so long-term outcomes of these diagnoses remain unknown.

To provide patients with better prognostic information, determine the risk of disease progression (here considered to be progression from less certain ARF through to RHD), and reflect on whether current management guidelines [3] are appropriate, the aim of this study is to describe patient outcomes after a diagnosis of possible or probable (collectively, ‘uncertain’) ARF, compared with definite ARF.

## Methods

### Study design and data sources

We used a retrospective cohort design and Cox proportional hazards regression to analyse the probability of disease progression after an initial diagnosis of ARF. Sub-analyses on initial ARF diagnoses with RHD outcome and adherence to penicillin adherence were conducted using multinomial logistic regression and generalised linear mixed-effects models (GLMM) respectively and are included as appendices. Deidentified data were extracted from the NT RHD Register (the Register) for a 5.5-year period (01/01/2013–30/06/2019). The Register includes all notified cases of ARF and RHD in the NT (approximately 3,500 individuals at the time of data extraction), with data on patient demographics, clinical features of ARF and RHD, medical appointments and secondary penicillin prophylaxis. Mortality data from the NT Births, Deaths and Marriages database are reviewed monthly and integrated with the Register.

### Study population and sample

The study population was defined as Indigenous Australians residing in the NT, which is approximately 74,546 people or 30% of the total NT population [13,14]. The Register contained information on 1,140 individuals with 1,567 diagnoses during the study period. Indigenous Australians residing in the NT who were registered with an initial diagnosis of possible, probable or definite ARF on the Register during the study period were included. Exclusions comprised non-Indigenous people (11; 1%) and cases whose first documented ARF episode was labelled a recurrence (14 possible ARF; 26 probable ARF; 176 definite ARF; total 216 or 19%). Non-Indigenous Australians were excluded due to potential differences in exposure to risk factors and access to health care, compared with Indigenous Australians. Those whose first documented episode was labelled a recurrence were assumed to have had a previous diagnosis of ARF before the study period began or in a different jurisdiction. The final cohort was 913 cases.

### Outcomes

The main outcome of interest was time to disease progression. Disease progression was defined as progression from possible to probable ARF, definite ARF or RHD; probable to definite ARF or RHD; or definite ARF to definite ARF recurrence or RHD at any time during the study period (Table 1). Individuals could progress and regress more than once during the study period, however only the first progression event during the study was counted.

**Table 1:** Definitions of disease progression.

Initial ARF diagnosis	Progression diagnosis
Possible ARF	Probable ARF, definite ARF or RHD
Probable ARF	Definite ARF or RHD
Definite ARF	Definite ARF recurrence or RHD

Disease severity outcomes were also calculated. The first of these was the proportion of those without baseline carditis who progressed to RHD. Secondly, the risk to develop mild or moderate to severe RHD compared to no RHD at initial ARF diagnosis. Definitions of disease severity were pre-defined by the 2020 Australian Guidelines and recorded as such in the Register.

Adherence to secondary prophylaxis with benzathine benzylpenicillin (BPG) injections was calculated as the percentage of received doses / prescribed doses x100. BPG injections are usually required once every 28 days for ARF secondary prophylaxis, i.e. 13 doses per year, but may be prescribed every 21 days, i.e. 17 doses per year, in more severe or breakthrough cases. The number of prescribed doses was estimated by dividing the number of days that BPG was prescribed (last dose date minus first dose date) divided by 21 or 28 for 3-weekly and 4-weekly BPG respectively. There is no agreed minimum time frame for adherence calculations for BPG; adherence calculations were restricted to cases that had been prescribed at least 6 doses of BPG (equivalent to 168 days for a 4-weekly regimen) in the interest of statistical robustness and consistency with previous studies [12]. Individuals with less than 168 days of prescribing time were coded as ‘not recorded’ and those who received oral antibiotics were excluded from adherence calculations due to the different effectiveness of oral penicillin compared to BPG injections [15]. Adherence was considered good if ≥80% of injections were received, in line with the 2020 Australian Guidelines [3].

#### Predictors

Predictors used in the analyses included initial ARF diagnosis, sex, age group (0–4, 5–14, 15–24, ≥25 years), clinical manifestations, and adherence to secondary prophylaxis. Initial ARF diagnosis was defined as the first diagnosis of possible, probable or definite ARF on the Register. Clinical manifestations were grouped as major manifestations (carditis; arthritis; Sydenham’s chorea; and subcutaneous nodules and erythema marginatum), minor manifestations (fever; elevated erythrocyte sedimentation rate (ESR) or C-reactive protein (CRP); prolonged P-R interval on electrocardiogram (ECG)), and evidence of group A streptococcal infection (elevated serological titre or cultured from throat swab) as per the 2020 Australian Guidelines [3].

### Statistical analysis

Descriptive statistics were used to describe the demographic and clinical characteristics at initial ARF diagnosis (possible, probable, definite). To investigate the associations between demographic and clinical characteristics with initial diagnostic category, a chi-square test for independence was performed. Kaplan-Meier curves and log rank test were used to assess the probability of survival free of disease progression and the cumulative incidence risk at each year since initial diagnosis was calculated.

Cox proportional hazards regression was used to determine whether time to disease progression differed according to ARF diagnosis category (definite, probable, possible), and whether it was predicted by age group at diagnosis, sex, adherence to secondary prophylaxis or clinical manifestations (joint manifestation-only and joint manifestation with carditis and/or chorea) at diagnosis. Cases who had a disease-free survival time of zero (n = 180 concurrent or ‘fulminant’ ARF and RHD diagnoses) were excluded from analyses of disease progression. Fulminant cases represent severe ARF which has already progressed to RHD at the time of first echocardiogram, or ARF occurring in someone with previously unrecognised latent RHD, or documentation of rheumatic carditis in the register as RHD (since distinguishing rheumatic carditis from established RHD is not always possible, and misclassification may occur during clinical reporting or data entry) [3]. In addition, cases with less than 168 days prescribing time (n = 138) were excluded from the analysis. The sample size for the Cox regression was 595 cases. Hazard ratios, 95% CI and p-values are reported.

A multinomial logistic regression model was performed to assess whether ARF diagnosis type (definite, probable, possible) was associated with RHD outcome (no RHD, mild RHD moderate-severe RHD). Moderate and severe RHD were combined to increase observations per group level. Predictors included age group, sex, adherence and clinical manifestations (carditis-only or joint manifestations-only. Chorea-only was excluded due to the small number of observations). Multinomial logistic regression was also used to assess associations between clinical manifestations (major criteria) and ARF diagnosis. Relative risk ratios, 95% confidence intervals (CI) and p-values were calculated for clinical manifestations, age at diagnosis and sex, using definite ARF as the reference group. Cases with less than 168 days prescribing time (n = 152) were excluded from analyses of RHD severity to give a final cohort of 761 cases.

**Fig 1:**
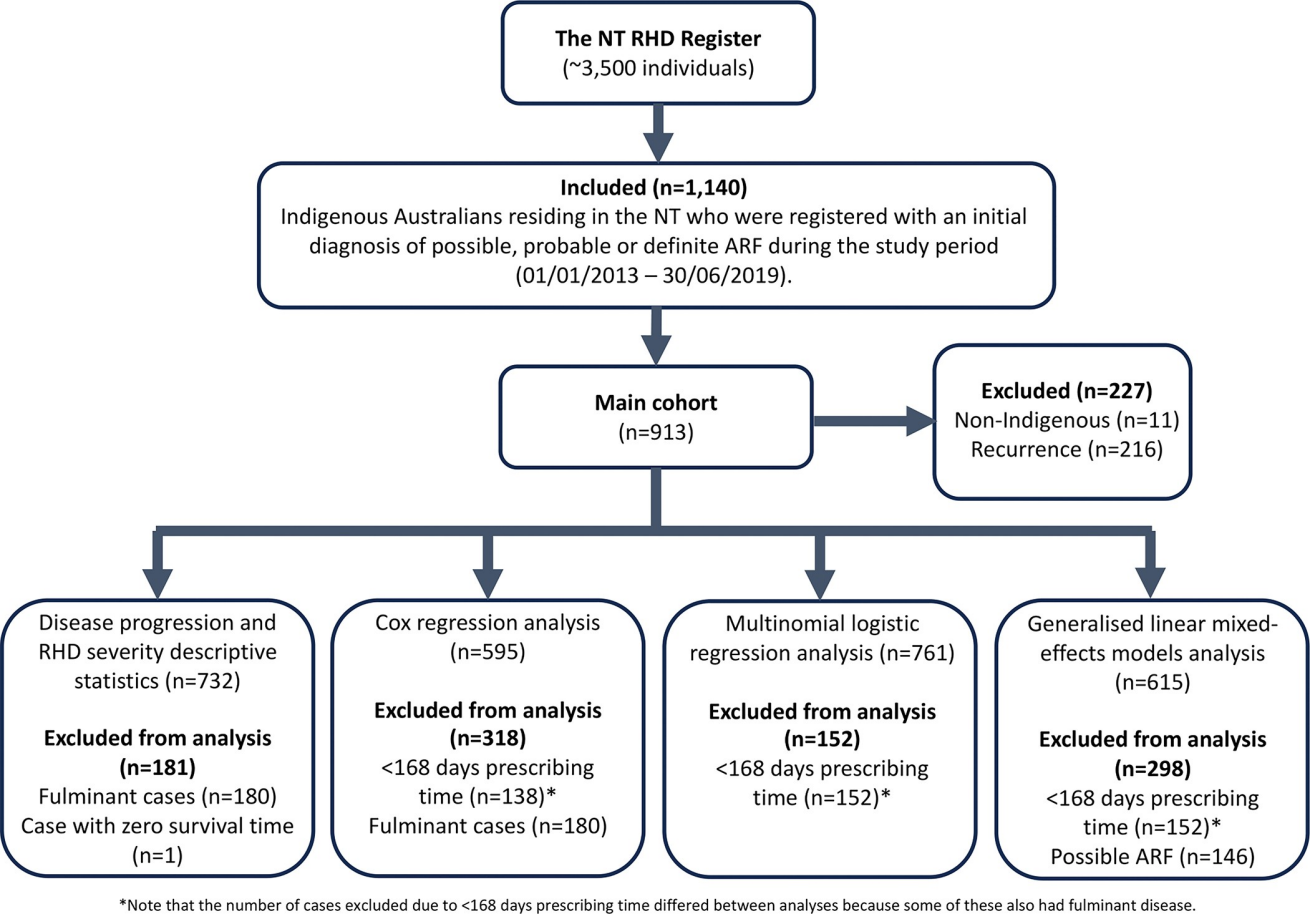
Study flow chart.

A GLMM (beta regression family) was developed to assess any differences in the long-term penicillin adherence between ARF diagnosis categories and examine longitudinal trends in adherence (glmmTMB package in R). This took into account the number of years receiving BPG prophylaxis and a random intercept for patient identification. Model residuals were checked for lack of patterns across fitted values and predictors (DHARMa package) and no temporal autocorrelation. Those who had less than 168 days of prescribing time (n = 152) were excluded from the analysis, as were those who had a diagnosis of possible ARF (n = 146), since secondary prophylaxis is only recommended for one year for possible ARF cases. The final cohort was 615 cases (Fig 1).

A p-value of ≤ 0.05 was considered significant in all analyses. Data were analysed using STATA version 15.1 (STATA Corp, College Station, TX), R (v4.1.3; R Project for Statistical Computing, Vienna, Austria) and Microsoft Excel (2016).

### Ethics approval

Approval was granted by the Human Research Ethics Committee of the Northern Territory Department of Health and Menzies School of Health Research (2019–3482) and registered with the University of Melbourne’s Medicine and Dentistry Human Ethics Sub-Committee (1955194). Consent was not required from study participants or participants’ parents/guardians due to the anonymous and non-interventional nature of this study. Access to register data was approved by the data custodian, Top End Health Service, NT Department of Health.

## Results

### Demographic and clinical characteristics

During the study period (01/01/2013–30/06/2019) there were 913 initial diagnoses of ARF among eligible register participants: 509 (56%) definite, 196 (21%) probable and 208 (23%) possible ARF. There were 3 deaths during the study period; one with an initial diagnosis of possible ARF and no reported progression, one with definite ARF and no reported progression, and one with definite ARF and RHD diagnosed simultaneously. All ARF diagnostic categories were more common among females than males and in those aged 5–14 years compared with other age groups (Table 2). Chi-square tests indicated there was no significant association between sex and ARF diagnostic category (χ^2^ = 0.6, df = 2, p = 0.72) but that there was a significant association between age group and ARF diagnostic category (χ^2^ = 20.4, df = 6, p = 0.002) (Table 2).

**Table 2:** Characteristics at initial acute rheumatic fever diagnosis among Indigenous Australians, Northern Territory, 2013–2019.

Characteristic	ARF						Total	
	Possible		Probable		Definite			
	n	(%)	n	(%)	n	(%)	n	(%)
Cases	208	(100)	196	(100)	509	(100)	913	(100)
Sex								
Male	97	(47)	87	(44)	243	(48)	427	(47)
Female	111	(53)	109	(56)	266	(52)	486	(53)
Age group (years)								
0–4	25	(12)	11	(6)	27	(5)	63	(7)
5–14	113	(54)	92	(47)	298	(59)	503	(55)
15–24	37	(18)	53	(27)	107	(21)	197	(22)
≥25	33	(16)	40	(20)	77	(15)	150	(16)
Median, IQR	10	6–19	14	9–22	12	9–18	15	8–19
Year of diagnosis								
2013	9	(4)	13	(7)	56	(11)	78	(9)
2014	13	(6)	11	(6)	72	(14)	96	(11)
2015	18	(9)	28	(14)	77	(15)	123	(13)
2016	40	(19)	36	(18)	81	(16)	157	(17)
2017	38	(18)	52	(27)	87	(17)	177	(19)
2018	58	(28)	40	(20)	95	(19)	193	(21)
2019*	32	(15)	16	(8)	41	(8)	89	(10)
Adherence (%)								
<50	16	(8)	22	(11)	52	(10)	90	(10)
50–79	47	(23)	57	(29)	120	(24)	224	(25)
80–99	47	(23)	55	(28)	164	(32)	266	(29)
≥100	36	(17)	36	(18)	109	(21)	181	(20)
Not recorded †	62	(30)	26	(13)	64	(13)	152	(17)
Clinical manifestation ‡								
Major								
Carditis	6	(3)	14	(7)	167	(33)	187	(20)
Joint	172	(83)	182	(93)	433	(85)	787	(86)
Sydenham chorea	3	(1)	0	(0)	62	(12)	65	(7)
Skin	2	(1)	1	(1)	7	(1)	10	(1)
Minor								
Fever	51	(24)	60	(31)	340	(67)	451	(49)
Elevated ESR or CRP	12	(14)	9	(6)	11	(2)	32	(5)
Prolonged P-R interval on ECG	10	(5)	19	(10)	196	(39)	225	(25)
Other								
Elevated GAS serological titre	183	(88)	168	(86)	480	(94)	831	(91)
GAS cultured from throat swab	5	(2)	7	(4)	18	(4)	30	(3)
Not recorded	6	(3)	1	(1)	0	(0)	7	(1)

ARF = acute rheumatic fever; GAS = group A beta haemolytic streptococcus; ESR = erythrocyte sedimentation rate; CRP = C-reactive protein; ECG = electrocardiogram; CM = clinical manifestation.

Note

* Only 6 months of data are available for 2019.

† Those where prophylaxis prescribing time was <168 days, including one case who entered the study on the last day.

‡ Cases may have more than one clinical manifestation, therefore the total does not add up to 100%.

¥ Elevated GAS serological titre or GAS cultured from throat swab only.

**Fig 2:**
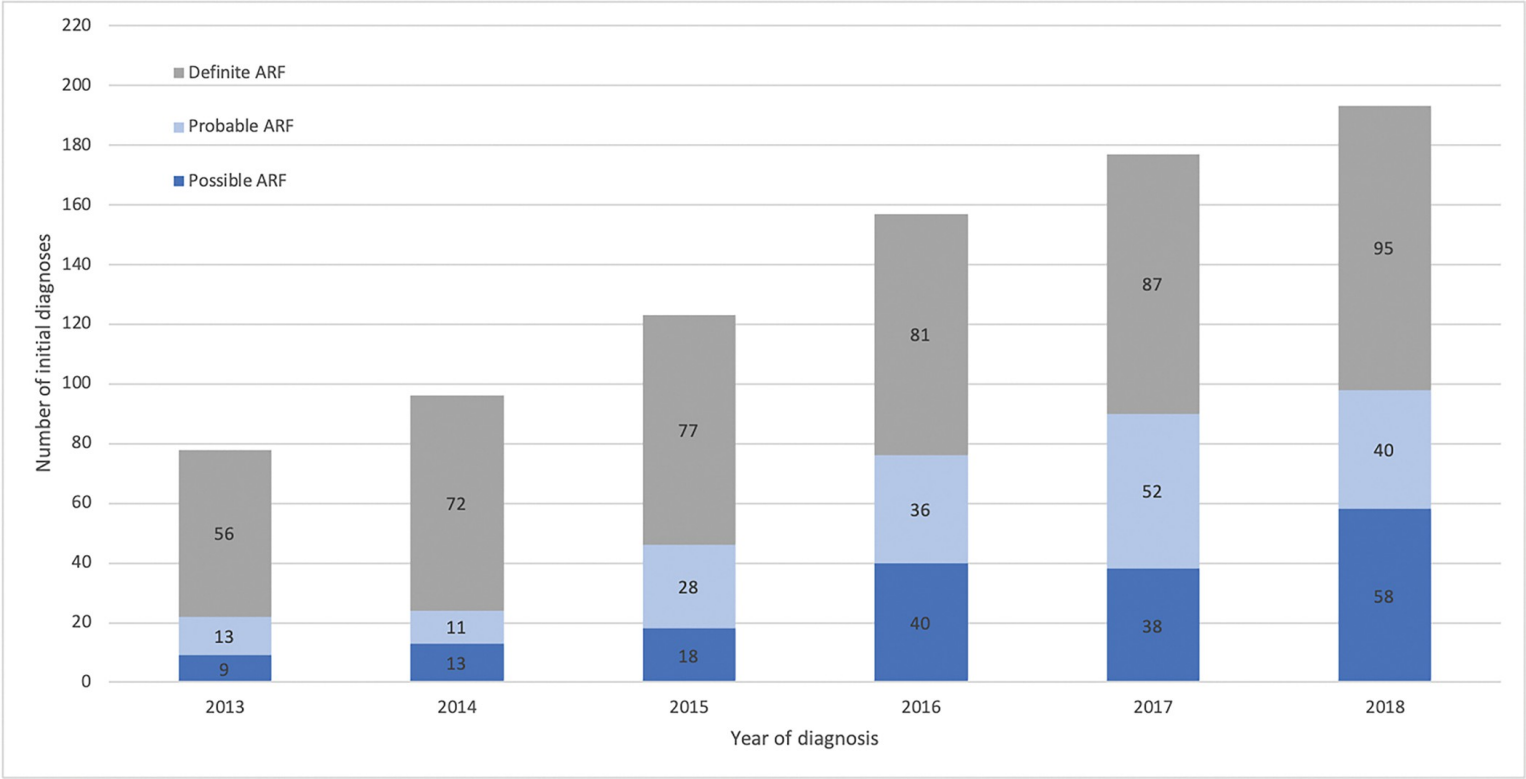
Number of acute rheumatic fever diagnoses among Indigenous Australians, Northern Territory, 2013–2018.

Each year, the total number of ARF diagnoses increased (Table 2). The proportion of uncertain diagnoses (possible or probable ARF) increased from 22/78 (28%) in 2013, when these diagnostic categories started to be recorded, to 98/193 (51%) in 2018 (Fig 2). Year of ARF diagnosis was significantly associated with ARF diagnostic category (χ^2^ = 50.5, df = 12, p = <0.001).

Joint pain was the most common major Jones criterion for all ARF diagnostic categories (Table 2). Cases presented with no evidence of streptococcal infection in 2% of cases classified as definite ARF, and in approximately 10% of possible and probable ARF cases [3]. Clinical manifestations were significantly associated with ARF diagnostic category (χ^2^ = 242, df = 18, p = <0.001). Penicillin adherence increased with ARF diagnosis certainty (Table 2): the proportion receiving ≥80% of scheduled BPG injections was 37%, 46%, and 56% among possible, probable and definite cases ARF respectively. However, the proportions with unknown adherence differed by ARF diagnostic category and was highest in those with possible ARF at 30%. Adherence was significantly associated with ARF diagnostic category (χ^2^ = 38.0, df = 8, p = <0.001).

### Disease progression

**Fig 3:**
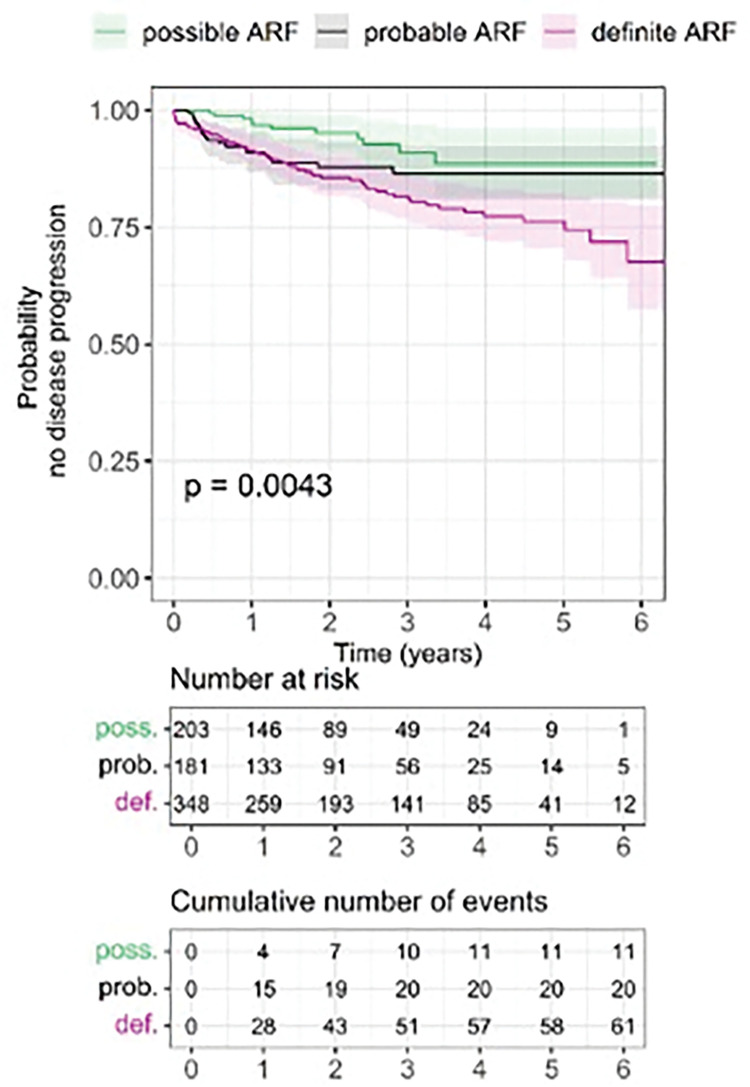
Kaplan-Meier progression-free survival estimates by initial acute rheumatic fever diagnosis among Indigenous Australians, Northern Territory, 2013–2019.

After excluding cases with zero disease-free survival time (n = 181), a total of 92 (13%) cases experienced disease progression during the study period. Of those with an initial diagnosis of definite ARF, 61/348 (18%) experienced disease progression, compared to 20/181 (11%) with probable ARF and 11/203 (5%) with possible ARF. Of those who progressed, 37/61 (61%) with definite ARF, 10/20 (50%) with probable ARF, and 5/11 (45%) with possible ARF (total of 52/92 or 57%) were diagnosed with RHD during the study period. Overall, progression to RHD was reported in 37/348 (11%) definite ARF, 10/181 (6%) probable ARF, and 5/203 (2%) possible ARF. The probability for disease progression significantly differed between possible, probable and definite ARF (p = 0.0043; log rank test; Fig 3). The Kaplan-Meier curves indicated the highest probability for no progression was for possible ARF, closely followed by probable ARF, while definite ARF had a lower probability (particularly beyond three years since diagnosis).

The probability curves and cumulative incidence risks of disease progression also indicated a continued average increase in cumulative risk of disease progression each year up to 4 and 3 years since initial diagnosis respectively for possible and probable ARF (Fig 3, Table 3). In contrast, the cumulative risk of disease progression continued to increase each year of the follow-up period for definite ARF.

**Table 3:** Cumulative incidence risk of disease progression by year of follow-up among Indigenous Australians, Northern Territory, 2013–2019.

Year	Possible ARF	Probable ARF	Definite ARF
	%, CI (95%)	%, CI (95%)	%, CI (95%)
1	2.46 (0.93–6.44)	9.03 (5.54–14.55)	8.66 (6.05–12.32)
2	4.85 (2.31–10.03)	12.13 (7.87–18.45)	14.43 (10.87–19.02)
3	9.05 (4.72–17.00)	13.54 (8.78–20.59)	17.98 (13.83–23.19)
4	11.44 (5.97–21.33)	13.54 (8.78–20.59)	22.19 (17.22–28.33)
5	11.44 (5.97–21.33)	13.54 (8.78–20.59)	23.26 (18.01–29.73)
5.5	11.44 (5.97–21.33)	13.54 (8.78–20.59)	33.55 (23.61–46.21)

Note

1. Only six months of data are available for 2019.

Sample size for this analysis is 732 cases (181 cases with zero survival time were excluded, including one case who entered the study on the last day).

Cox proportional hazards regression indicated that disease progression was 2.19 times more likely in those with definite ARF than those with possible ARF (p *=* 0.026) (Table 4).

**Table 4:** Cox proportional hazards analysis of predictors associated with disease progression among Indigenous Australians, Northern Territory, 2013–2019.

Variable	Hazard Ratio	Confidence Interval (95%)	P value
**ARF Diagnosis**			
Possible ARF	Reference		
Probable ARF	1.44	0.66–3.15	0.353
Definite ARF	2.19	1.10–4.34	**0.026**
**Sex**			
Male	Reference		
Female	1.06	0.67–1.67	0.811
**Age group (years)**			
0–4	0.73	0.26–2.06	0.552
5–14	Reference		
15–24	1.28	0.75–2.20	0.366
≥25	1.71	0.93–3.15	0.084
**Adherence**			
<50%	0.31	0.09–1.11	0.072
50–79%	1.48	0.66–3.29	0.337
80–99%	Reference		
≥100%	2.22	0.86–5.78	0.100
**Clinical manifestations**			
Carditis and/or chorea with or without joint manifestation	Reference		
Joint manifestation only (i.e. no carditis, no chorea)	0.48	0.24–0.95	**0.035**

ARF = acute rheumatic fever.

Note

1. Only six months of data are available for 2019.

2. Sample size for this analysis is 595 cases (138 cases with <168 days prescribing time and 180 cases with zero survival time were excluded)

3. Proportional hazard assumptions were not met for drug adherence nor joint manifestation and therefore, an interaction term with linear time was fitted for these 2 factors (likelihood ratio test Chi2(3) = 14.9; Chi2(1) = 7.4, P<0.01 for both). The time varying effect for joint manifestation was 1.7 (P = 0.012) and 2.0 for prophylaxis adherence of <50% (P = 0.010), both indicating a weakening protective effect with time.

The analysis also indicated that having joint manifestations only at initial diagnosis of ARF was protective, with this group being 52% less likely to experience disease progression compared with those who had other clinical manifestations including carditis and/or chorea at initial diagnosis and with or without joint manifestation (p = 0.035) (Table 4). Though not statistically significant, disease progression was equally likely between sexes and age groups. In this dataset, the hazard ratios of cases experiencing disease progression increased with increasing adherence, but this association was not statistically significant (Table 4).

### Risk of developing rheumatic heart disease

The proportion of those who had no baseline carditis but still progressed to RHD (at any time during the study) is presented by clinical manifestation (joint-only or chorea-only) in S1 Text. The multinomial logistic regression model demonstrated a significantly higher risk of progression from no RHD to RHD if the initial diagnosis was definite compared to possible ARF (p<0.001 for both mild and moderate-severe RHD outcomes) (Table 5).

**Table 5:** Multinomial logistic regression of initial acute rheumatic fever diagnosis and development of rheumatic heart disease, by severity of rheumatic heart disease, among Indigenous Australians, Northern Territory, 2013–2019.

Multinomial model	Relative Risk Ratio	Confidence Interval (95%)	P value
Risk to develop mild RHD compared to no RHD after an initial ARF diagnosis			
**ARF diagnosis**			
Possible ARF	Reference		
Probable ARF	2.27	0.91–5.64	0.078
Definite ARF	6.39	2.85–14.35	**<0.001**
**Age group**			
< = 4 yrs	0.29	0.07–1.26	0.098
5–14 yrs	Reference		
15–24 yrs	1.71	1.04–2.81	**0.034**
> = 25 yrs	1.98	1.11–3.54	**0.021**
**Clinical Manifestations**			
Carditis only–Yes	0.95	0.27–3.32	0.939
Joint only–Yes	0.49	0.32–0.76	**0.002**
**Adherence**			
<50%	0.49	0.24–1.01	0.054
50–79%	0.77	0.47–1.26	0.294
80–99%	Reference		0.591
≥100%	0.86	0.50–1.48	
**Gender**–Female			0.084
Risk to develop moderate to severe RHD compared to no RHD after an initial ARF diagnosis			
**ARF diagnosis**			
Possible ARF	Reference		
Probable ARF	2.51	0.46–13.72	0.289
Definite ARF	13.92	3.22–60.16	**<0.001**
**Age group**			
< = 4 yrs	1.80	0.64–5.04	0.261
5–14 yrs	Reference		
15–24 yrs	1.88	0.98–3.61	0.059
> = 25 yrs	2.11	0.94–4.72	0.071
**Clinical Manifestations**			
Carditis only–Yes	3.14	1.11–8.82	**0.030**
Joint only–Yes	0.19	0.11–0.34	**<0.001**
**Drug adherence**			
<50%	0.20	0.06–0.74	**0.015**
50–79%	0.67	0.34–1.31	0.237
80–99%	Reference		
≥100%	1.41	0.75–2.66	0.281
**Gender**–Female	0.90	0.52–1.55	0.710

ARF = acute rheumatic fever; RHD = rheumatic heart disease.

Note

1. Only six months of data are available for 2019.

2. Sample size for this analysis is 761 cases (152 cases with <168 days prescribing time were excluded).

There was a strong protective effect for those with joint manifestations only, with a 51% reduced risk to progress to mild RHD and an 81% reduced risk to progress to moderate or severe RHD from no RHD, with all other covariates held constant (p<0.001 for both; Table 5). There was a three-fold increased risk of developing moderate-severe RHD compared to no RHD for patients with carditis-only clinical manifestation (p = 0.030; Table 5).

### Penicillin adherence

Adherence to secondary penicillin prophylaxis was significantly (p = 0.028) higher among patients with definite ARF compared with probable ARF (S2 Text).

## Discussion

We show that the vast majority of children and young people diagnosed with possible ARF (95%) and probable ARF (89%) did not progress to definite ARF or RHD. These findings highlight that definite, probable and possible ARF represent three distinct diagnostic categories with different prognoses. The international guidelines known as the Revised Jones Criteria currently only articulate two categories: definite and probable ARF [6]. While many people labelled with possible ARF in our dataset may never have had true ARF, is not entirely benign; 4/200 (2%) with an initial diagnosis of possible ARF and a normal echocardiogram developed RHD; 3 within 12 months (1.5%), and 1 within 24 months (0.5%). RHD may have been evolving from the time of initial presentation but criteria were not met at initial presentation for a diagnosis of definite or even probable ARF. The inability to predict which individuals are the ones most likely to progress highlights the need for follow-up echocardiography, and development of biomarkers to diagnose and risk-stratify ARF. More research in this domain is needed [16]. Current Australian guidelines recommend 12 months of secondary prophylaxis after a diagnosis of possible ARF [3]. Our findings support this approach, since development of RHD more than 12 months after ‘possible ARF’ appears to be no more common than the background population RHD risk, based on RHD population prevalence of 2% in 5–9 year old children undergoing echocardiographic screening, and 6.8% in 10–15 year olds, in a high-burden NT setting [17].

Healthcare providers appear to be increasingly confident in applying ‘probable’ and ‘possible’ ARF diagnoses since 2013 when these were defined and introduced into the NT register. When total ARF diagnoses are captured, our data show that the increases over time chiefly represent improving detection of uncertain cases from 22/78 (28%) in 2013 to 98/193 (51%) in 2018, with a smaller proportional increase in numbers of definite ARF (Fig 2). Efforts to improve detection have included promotion of accessible clinical practice guidelines [2,3,18] and a diagnosis calculator available for smartphone use [19].

The frequency of ARF clinical manifestations reported in our study are similar to previous NT data, [20] showing arthritis to be the most common major criterion followed by carditis then chorea, with almost all additionally having fever [21]. Rheumatic carditis detected on echocardiogram is diagnostic of definite ARF and should not be classified as possible or probable ARF. Possible and probable ARF cases noted to have echocardiographic valvar disease in this study may have had known RHD and presented with an acute illness not meeting criteria for definite ARF. When a child with RHD presents with fever or elevated inflammatory markers, distinguishing ARF from other febrile conditions with underlying RHD can be difficult and may result in a ‘possible’ or ‘probable’ ARF diagnosis; the child is already prescribed penicillin prophylaxis but a new ARF diagnosis may alter both the interval and duration of prophylaxis depending on their age. Sydenham chorea is synonymous with ARF; once alternative causes of chorea have been excluded, a child with chorea (especially if from a high-risk setting such as the NT) receives a diagnosis of definite ARF. The possible and probable cases in this study noted to have chorea may, for instance, have had a transient movement disorder not clearly evident as Sydenham chorea.

The cumulative incidence risk of disease progression per year for those diagnosed with possible ARF stopped increasing at 11%, 4 years from initial diagnosis, and for those diagnosed with probable ARF at 14%, 3 years from initial diagnosis. In contrast, the cumulative incidence risk of disease progression after definite ARF continued to increase each year from initial diagnosis, up to 34% at 5.5 years from initial diagnosis (Table 3). While the trend of increasing risk of disease progression from initial diagnosis largely concurs with previous studies, the cumulative incidence risk of progression to RHD after definite ARF was lower in our study compared with others in the same setting [11,22]. This may reflect exclusion of the most-severe ARF cases from cumulative incidence calculations in our study, since they already had the outcome of interest (RHD) at baseline. Other explanations may include improving delivery of secondary prophylaxis which occurred during our study period [23], or greater clinical detection over time of more subtle ARF cases which are less likely to progress, which the increasing ARF detection rates reported here suggest.

Cox proportional hazards analysis demonstrated that those with probable and definite ARF were more likely to experience disease progression (compared to those with possible ARF). The risk of progression from no RHD to RHD was significantly higher if the initial diagnosis was definite ARF compared to possible ARF (Table 5). Those who had only joint manifestations at initial diagnosis of ARF were 52% less likely to experience disease progression (compared to carditis and/or chorea) (Table 4). Joint-only manifestations, which may only fulfill criteria for possible or probable ARF, may in other settings be labelled as ‘post streptococcal reactive arthritis’, considered to be a separate entity from ARF [24,25]. However, since 6.6% (37/560) of people still developed RHD in our series after initial joint-only presentations, we consider a diagnosis of ARF and provision of secondary prophylaxis to be safer in the NT context, where there is a high burden of disease.

In this dataset, of the cases that progressed (and had 168 days or greater prescription time), less than half (40/86; 47%) were adherent to ≥80% of their BPG injections and adherence for both definite and probable ARF cases was shown to decline over time from initial diagnosis (Fig 3). These findings are consistent with trends identified in previous research using data from the NT RHD Register, which found that adherence improved between 2007 and 2013 (19% of people were adherent in 2007 compared with 32% in 2013) and that increasing time since first diagnosis was associated with lower adherence [12]. Furthermore, we found that people with ARF who had greater penicillin adherence were also those with a higher risk of disease progression—but this association was not statistically significant (Table 4). These findings may be attributable to the association between disease severity and adherence, delays in presentation and referral of patients, or other factors. Previous research using the same NT register, but an earlier time period, identified that those with most severe disease (e.g. requiring surgery) were those who were subsequently most adherent, potentially attributable to higher-level health system supports implemented for these high-risk individuals, and greater patient understanding of the risks of non-adherence [12]. The same paper showed a clear protective effect of adherence on ARF outcomes when adherence prior to the event of interest was examined. Therefore, we conclude that adherence reduces ARF recurrences or RHD progression, but in this dataset, we were unable to demonstrate this relationship. Also, we used percent adherence which does not necessarily factor in ‘days at risk’ [12]; that is, the timing of dose administration.

A strength of this study is that ARF is notifiable in the NT and therefore ARF diagnoses are well captured and documented in the NT RHD Register. However, despite increasing detection and reporting of possible and probable ARF cases as shown in this paper, missed diagnoses of ARF are still likely to occur, with up to 75% of newly diagnosed cases of RHD in northern Australia having no previous diagnosis of ARF [3,26]. A limitation of this study is the short follow up time of 5.5 years, but since the risk of disease progression appeared to stabilise approximately for possible and probable ARF 4 years from initial diagnosis the difference in outcomes compared with definite ARF was able to be demonstrated. As noted, concurrent ARF and RHD diagnoses were excluded since they had a disease-free survival time of zero so could not be included in analysis of progression. Exclusion of these most severe ARF cases impacted sample size and statistical rigour, follow-up time and the ability to calculate median survival time, an important factor in the interpretation of survival data, however they were accounted for in the multinomial model [27]. Finally, an analysis of echocardiogram data could have added further context and value to the study. Given that in some patients, signs of carditis appear after presentation of ARF (usually within the first two to six weeks), echocardiogram data collected within the acute phase of illness may have identified additional carditis and affected diagnostic classification, potentially leading to an overestimate of the risk of disease progression in our study. However, the analyses and conclusions represent the reality of current clinical practice in our setting, characterised by vast geographic distances to healthcare with challenges in obtaining timely follow-up echocardiograms. An analysis of ARF cases, by diagnostic category, who had follow-up echocardiograms after the acute phase of illness would also add to the assessment of mid and long-term disease progression, and should be considered in future research.

## Conclusion

This study provides much needed data about the likelihood of disease progression for children and young people given a diagnosis of possible and probable ARF. Children and their families can be reassured that possible ARF, probable ARF and ARF affecting the joints only, carry relatively good prognoses. These diagnoses are more consistent with post-streptococcal reactive arthritis than true ARF. However, secondary prophylaxis with penicillin is still of critical importance given the morbidity of recurrences and the small but present risk of progression to RHD. For definite ARF, our study confirms findings from previous research, in that the proportion of cases that experience disease progression increases over time after a first episode, and we add new data demonstrating that the number of children and young people diagnosed with definite ARF in the NT has continued to increase each year, despite the addition of new diagnostic categories in 2013.

## Supporting information

S1 Text Risk of developing rheumatic heart disease. (DOCX)

S2 Text Penicillin adherence. (DOCX)
