# Supplementary material for: Outcomes of possible and probable rheumatic fever: A cohort study using northern Australian register data, 2013–2019
Source: PLOS Glob Public Health. 2024 Jan 3;4(1):e0002064. doi: 10.1371/journal.pgph.0002064 (PMC10763935; doi:10.1371/journal.pgph.0002064)
Supplement: S1 Text — (DOCX) [file pgph.0002064.s001.docx]

### S1 Text: Risk of developing rheumatic heart disease

The proportion of those who had no baseline carditis but still progressed to RHD (at any time during the study) is presented by clinical manifestation (joint-only or chorea-only) in Supplementary Table 1. Of those who progressed to RHD with joint-only manifestations, 46% (17/37) progressed *after one year*. Of those who progressed to RHD with chorea-only manifestations, only 3% (1/30) progressed *after one year*. An additional case with neither chorea nor joint manifestations also progressed to RHD *after one year*, bringing the total cases with progression to RHD *after one year* to be 19 cases.

**Table A: Progression to rheumatic heart disease by clinical manifestation among Indigenous Australians, Northern Territory, 2013-2019**

| ARF diagnosis | Joint-only with no baseline echocardiographic pathology | | Chorea-only with no baseline echocardiographic pathology | |
| --- | --- | --- | --- | --- |
|  | Total cases N | Progression to RHD n (%) | Total cases N | Progression to RHD n (%) |
| Possible ARF | 167 | 4 (2%) | 2 | 0 (0%) |
| Probable ARF | 164 | 10 (6%) | 0 | - |
| Definite ARF | 229 | 23 (10%) | 28 | 3 (11%) |
| All ARF / total | 560 | 37 (7%) | 30 | 3 (10%) |
