# Supplementary material for: Outcomes of possible and probable rheumatic fever: A cohort study using northern Australian register data, 2013–2019
Source: PLOS Glob Public Health. 2024 Jan 3;4(1):e0002064. doi: 10.1371/journal.pgph.0002064 (PMC10763935; doi:10.1371/journal.pgph.0002064)
Supplement: S2 Text — (DOCX) [file pgph.0002064.s002.docx]

### S2 Text: Penicillin adherence

The GLMM estimated that patients with definite ARF had a significantly higher adherence to penicillin prophylaxis compared with probable ARF (p=0.028). Possible ARF, requiring only 12 months of treatment, was excluded from adherence comparisons. Time on prophylaxis was also significantly inversely associated with adherence, with adherence being higher in the first year after diagnosis and falling in years 2 to 6 after ARF diagnosis, for both definite and probable ARF (p<0.001 for all).


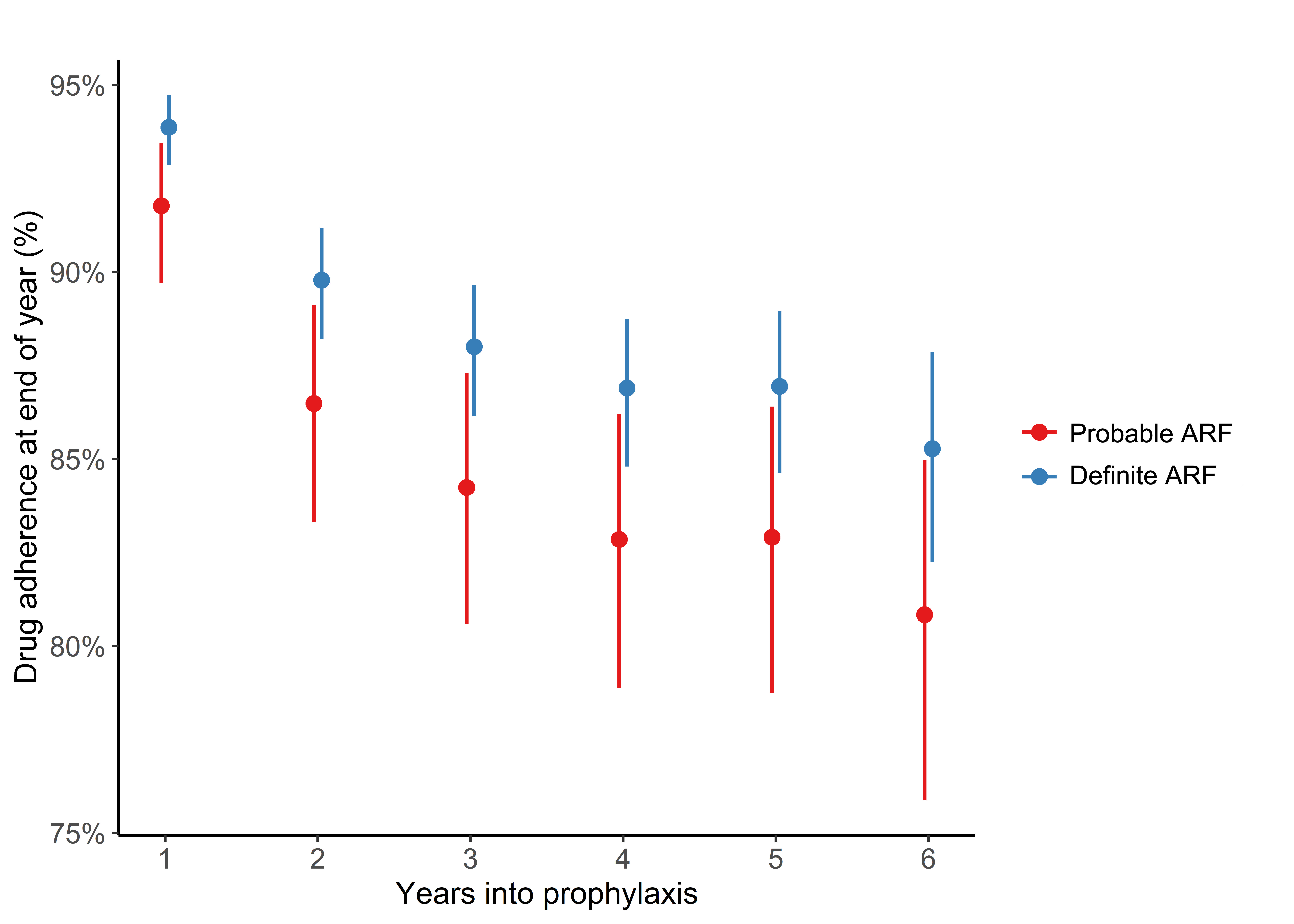


**Fig A. Penicillin adherence by initial acute rheumatic fever diagnosis and year since diagnosis among Indigenous Australians, Northern Territory, 2013-2019.**
